# The validity of clinicians’ diagnoses: Is it bread and butter?

**DOI:** 10.1192/j.eurpsy.2022.158

**Published:** 2022-09-01

**Authors:** A. Stevens

**Affiliations:** University of Tübingen / Medizinisches Begutachtungsinstitut, Medicolegal Assesmment, Tubingen, Germany

**Keywords:** Major depression, diagnostic accuracy, embitterment, response bias

## Abstract

Major depression has become one of the most frequent diagnoses in Germany. It is also quite prominent in cases referred for medicolegal assessment in insurance, compensation or disability claims. This report evaluates the validity of clinicians’ diagnoses of major depression in a sample of claimants. In 2015, n = 127 consecutive cases were examined for medicolegal assessment. All had been diagnosed with major depression by clinicians. All testees underwent a psychiatric interview, a physical examination, they answered questionnaires for depressive symptoms according to DSM-5, embitterment disorder, post-concussion syndrome (PCS) and unspecific somatic complaints. Performance and symptom validity tests were administered. Only 31% of the sample fulfilled the diagnostic criteria for DSM-5 major depression according to self-report, while none did so according to psychiatric assessment. Negative response bias was found in 64% of cases, feigned neurologic symptoms in 22%. Symptom exaggeration was indiscriminate rather than depression-specific. By self-report (i.e. symptom endorsement in questionnaires), 64% of the participants qualified for embitterment disorder and 93% for PCS. In conclusion, clinicians’ diagnoses of depression seem frequently erroneous. The reasons are improper assessment of the diagnostic criteria, confusion of depression with bereavement or embitterment and a failure to assess for response bias.

**Disclosure:**

No significant relationships.

